# Tissue distribution of 4-hydroxy-*N*-desmethyltamoxifen and tamoxifen-*N*-oxide

**DOI:** 10.1007/s10549-012-2074-9

**Published:** 2012-05-05

**Authors:** Jennifer Gjerde, Sara Gandini, Aliana Guerrieri-Gonzaga, Line L. Haugan Moi, Valentina Aristarco, Gunnar Mellgren, Andrea DeCensi, Ernst A. Lien

**Affiliations:** 1 Institute of Medicine, University of Bergen, Bergen, Norway; 2 Hormone Laboratory, Haukeland University Hospital, 5021 Bergen, Norway; 3 Division of Epidemiology and Biostatistic, European Institute of Oncology, Milan, Italy; 4 Division of Cancer Prevention and Genetics, European Institute of Oncology, Milan, Italy; 5 Department of Clinical Pathology, The University Hospital of North Norway, Tromsø, Norway; 6 Division of Medical Oncology, Galliera Hospital, Genoa, Italy

**Keywords:** Breast cancer, Tamoxifen, 4-OH-*N*-desmethyltamoxifen, Endoxifen, Tamoxifen-*N*-oxide, MCF-7

## Abstract

Tamoxifen dosage is based on the one-dose-fits-all approach. The anticancer effect of tamoxifen is believed to be due to the metabolites, 4-hydroxytamoxifen (4OHtam), and 4-hydroxy-*N*-desmethyltamoxifen (4OHNDtam/endoxifen). These demethylated metabolites of tamoxifen have been associated with its side effects, whereas the effect mediated by tamoxifen-*N*-oxide (tamNox) is still poorly understood. Our objective was to improve the therapeutic index of tamoxifen by personalizing its dosage and maintaining serum tamoxifen metabolite concentrations within a target range. We examined the levels of tamoxifen, 4OHtam, 4OHNDtam, *N*-desmethyltamoxifen (NDtam), *N*-desdimethyltamoxifen (NDDtam), and tamNox in serum and in breast tumors specimens of 115 patients treated with 1, 5 or 20 mg/day of tamoxifen for 4 weeks before surgery in a randomized trial. Furthermore, the metabolism of tamNox in MCF-7 breast cancer cells was also studied. The concentrations of tamoxifen and its metabolites in tumor tissues were significantly correlated to their serum levels. Tumor tissue levels were 5–10 times higher than those measured in serum, with the exception of tamNox. In MCF-7 cells, tamNox was converted back to tamoxifen. In contrast to the tissue distribution of tamNox, the concentrations of 4OHtam and 4OHNDtam in tumor tissues corresponded to their serum levels. The results suggest that implementation of therapeutic drug monitoring may improve the therapeutic index of tamoxifen. Furthermore, the tissue distribution of tamNox deviated from that of the other tamoxifen metabolites.

## Introduction

Tamoxifen, a selective estrogen receptor modulator (SERM), is used in the treatment of breast cancer patients with estrogen receptor (ER)-positive (+) tumors. It is the gold standard in adjuvant endocrine therapy for premenopausal women [1] and may also be used as a preventive agent in women with a high risk of breast cancer [2]. Whereas aromatase inhibitors (AI) have become the first-line adjuvant treatment for postmenopausal women, tamoxifen is still a treatment option in patients who experience adverse effects that make continuation of AI therapy clinically difficult [3].

Tamoxifen can act as an estrogen antagonist or agonist depending on tissue type. The agonistic effects of tamoxifen are associated with favorable effects, such as prevention of bone fractures, but also detrimental effects, including an increased risk of endometrial cancer and venous thromboembolic events [4].

The anticancer effect of tamoxifen is believed to be due to its metabolites, 4-hydroxytamoxifen (4OHtam), and 4-hydroxy-*N*-desmethyltamoxifen (4OHNDtam/endoxifen), with high affinity for the ER [5, 6]. The demethylated metabolites of tamoxifen have been associated with side effects [7], whereas the effects mediated by tamoxifen-*N*-oxide (tamNox) are still poorly understood.

The cytochrome P450 (CYP) enzyme CYP2D6, is crucial for the hydroxylation of tamoxifen to its active metabolites, 4OHtam, and 4OHNDtam. CYP2B6, 2C9, and 2C19 also hydroxylate tamoxifen [8–12]. Inactivation reactions involve sulfotransferases (SULTs) and uridine-5-diphospho-glucuronosyltransferases (UGTs). Concomitant medications that interact with these enzymes, including some selective serotonin reuptake inhibitors (SSRI), influence the serum levels of 4OHNDtam [13].

CYP2D6, CYP2C19, and SULT1A1 activities have been suggested as possible predictors of outcome during tamoxifen treatment [13–15]. Due to the multiplicity of factors that influence the pharmacokinetics of tamoxifen, it is not surprising that results from studies linking genetic polymorphisms of these enzymes to clinical outcomes during tamoxifen treatment have shown inconsistent results [16–19].

TamNox is generated by flavin-containing monooxygenase (FMO) [20]. Anti-estrogenic activity of tamNox has been observed. Bates et al*.* [21] have shown that the growth of the ER-positive cell line MCF-7 was inhibited to the same level by tamoxifen and tamNox. To date, neither its metabolism nor its effects have been extensively studied in humans. In liver microsomes, tamNox is rapidly converted back to tamoxifen [22], and may act as a reservoir for tamoxifen in tissues [23].

In a previous pre-surgical study, we used an HPLC method to examine the serum and tissue levels of tamoxifen and its metabolites after 4 weeks of treatment [24, 25]. 4OHNDtam and tamNox could not be analyzed in that study. We report here for the first time the tumor tissue distribution of 4OHtam, 4OHNDtam, and tamNox during normal dose- and low dose-tamoxifen treatment.

## Materials and methods

### Patients and study protocol

The study protocol and the main results have been described in detail elsewhere [24]. The study population consisted of 120 subjects, both pre- and postmenopausal women of a median age of 60 years with newly diagnosed ER+ and/or progesterone receptor (PR+) breast tumors <5 cm in diameter and with no sign of distant metastasis. The study was conducted under the approval of EIO Ethic Committee and all patients provided written consent to their participation in the study. The samples for this study were collected from the European Institute of Oncology (Milan). A total of 120 subjects with ER+ and/or PR+ cancers were randomly assigned to a conventional regimen of 20 mg tamoxifen daily or a bolus dose of 20 mg tamoxifen on day 1, followed by 1 or 5 mg daily for a total of 28 days before surgery. As tamoxifen requires 4–6 weeks to reach steady-state levels in vivo [26], a bolus dose of 20 mg tamoxifen was introduced on day 1 to shorten the time required to reach steady-state levels. This also allowed steady-state drug levels to be reached within 4 weeks, even for patients who were randomly assigned to the lowest dose of tamoxifen [24].

### Sample collection

Biopsies from tumor were retrieved during surgery and were stored at −80 °C until used for high pressure liquid chromatography-tandem mass spectrometry (HPLC-MS/MS)-based measurements of tamoxifen and its metabolites. Blood samples were collected between 7 a.m. and 9 a.m at baseline and on the day of surgery, and were stored at −80 °C until analysis. All the samples had been thawed once for a previous study and were refrozen at −80 °C. A total of 63 tumor tissue samples and 115 serum aliquots were available for tamoxifen and metabolite analyses.

### Determination of tamoxifen and metabolite levels in serum and tissue

Tissue samples were prepared as described previously [27]. The samples were homogenized (1:5, w/v) in 50 mM Tris–HCl, pH 7.4, at 20,000 rev/min. The homogenate was mixed with an equal volume of 100 % acetonitrile and the resulting protein precipitate was removed by centrifugation. The supernatants were analyzed by HPLC-MS/MS. We did not observe a reduction of tamNox to tamoxifen during sample preparation (data not shown). Tamoxifen citrate and 4OHtam were purchased from Sigma-Aldrich (Steinheim, Germany), the internal standard, deuterated 5-tamoxifen (D5tam), and tamNox from BioChem (USA), and 4OHNDtam from Sintef Materials and Chemistry (Oslo, Norway). NDtam and NDDtam were gifts from Imperial Chemical Industries, PLC Pharmaceutical division (Macclesfield, UK). An HPLC-MS/MS system was used for the determination of tamoxifen and five of its metabolites in serum [28, 29].

### Cell culture

The ER+ human breast adenocarcinoma MCF-7 cells were cultured at 37 °C in 5 % CO_2_, in Dulbecco’s modified Eagle medium (DMEM, Invitrogen, Carlsbad, CA) containing 4.5 g/l glucose and supplemented with 10 % fetal bovine serum (Invitrogen), 1 % (v/v) penicillin/streptomycin solution, and 1 μM insulin. The cells were preconditioned in phenol red-free DMEM (Invitrogen, Carlsbad, CA) containing charcoal-stripped fetal bovine serum (Hyclone) and the above supplements, for 2 days. The cells were seeded in six-well plates at a density of 300,000 cells/ml and then treated with 0.2 mM estradiol (E2) and 80 ng/ml tamNox. The growth medium from the incubated cell cultures was collected and the concentrations of tamoxifen and its metabolites determined.

### Statistical analysis

Non-parametric Wilcoxon tests were used to investigate the differences in concentrations of metabolites measured in serum and breast cancer tissue between groups (1, 5, and 20 mg), and to examine differences in tissue-to-serum ratios. *P* < 0.05 was considered statistically significant. Two-tailed Spearman correlation rank tests were used to examine the correlations between tamoxifen and metabolite concentrations in serum and breast cancer tissues. The level of statistical significance for correlation analyses was set at *P* < 0.01 to account for multiple comparisons. All *P* values were two-sided. Statistical analyses were performed with SAS statistical software version 9.0 (SAS Institute Inc, Cary, NC) and/or SPSS software, version 14.0.2 (SPSS Inc, Chicago, Illinois).

## Results

Patient and tumor characteristics of all the subjects are summarized in Table 1. Sixty-three frozen ER+ tumor specimens were obtained during surgery. No significant differences were found at baseline among subjects in the treatment groups. Table 1 shows the mean number of menopausal related symptoms, such as sweating, night sweats, and hot flashes experienced by the patients at baseline.

**Table 1:** Patient and tumor characteristics at baseline

	Tamoxifen dose (mg/day)		
	1 mg (*n* = 39)	5 mg (*n* = 40)	20 mg (*n* = 37)
Serum samples, (*n*)	39	40	36^a^
Age (years)^b^	62 (50, 70)	62 (51, 71)	58 (51, 65)
Body mass index (kg/m^2^)^b^	25.5 (23.9, 29.1)	25.7 (22.3, 30.5)	26.0 (22.4, 28.3)
Pre/peri/postmenopausal (*n*)	9/1/29	10/1/29	8/1/28
Surgical specimen available (*n*)	25	19	19
Tumor size (mm)	21 (17, 25)	18 (15,26)	17 (14, 21)
Menopause related symptoms at baseline^c^			
Sweating, yes/no/missing	12/18/9	6/15/19	11/14/12
Night sweating, yes/no/missing	15/15/9	7/17/16	8/20/9
Hot flashes, yes/no/missing	15/17/7	7/17/16	7/20/10

^a^Serum sample was not available from one patient in the 20 mg dosing group

^b^Values are presented as medians with lower and upper quartiles (Q1, Q3)

^c^Missing data; data not filled in the Menopause-Specific Quality of Life questionnaire by the patients

The levels of tamoxifen and its metabolites in all serum and tissue samples are presented in Table 2. Tamoxifen serum levels showed a wide variation among subjects within each dosing group. We observed a dose-concentration relationship for tamoxifen and all its metabolites in serum and tissues samples (*P* < 0.001). In contrast to the tissue levels of tamoxifen, 4OHtam, 4OHNDtam, and NDtam, we did not observe any increase in the tissue levels of tamNox in parallel with increasing doses and increasing serum concentrations. A positive correlation was found between serum levels and the concentrations of tamoxifen and its metabolites in malignant breast tissue (*P* < 0.001; Table 3). Thus, the serum concentrations of the active hydroxylated tamoxifen metabolites give information about their concentrations in the target tissue. Interestingly, only the tumor tissue/serum ratio of tamNox decreased with increasing doses (*P* < 0.0001). This demonstrates that the tumor tissue distribution of tamNox deviates substantially from that of the other metabolites.

**Table 2:** Concentrations of tamoxifen and metabolites

	Dose	Serum		Tumor tissue		Ratio tissue/serum	
		Median (Q1, Q3) (ng/ml)	*P* ^a^	Median (Q1, Q3) (ng/g)	*P*	Median (Q1, Q3)	*P*
Tamoxifen	1	6.3 (4.4, 9.3)	<0.0001	56.1 (38.6, 101.1)	<0.0001	9.4 (5.7, 14.5)	0.35
	5	25.7 (19.1, 37)		216.2 (115.6, 401)		9.7 (5.5, 17)	
	20	82.1 (69.7, 106.9)		631.2 (249.4, 1077.2)		7.1 (3.5, 12)	
4OHtam	1	0.3 (0.3, 0.4)	<0.0001	4.5 (2.8, 7.9)	<0.0001	18.1 (7.7, 28.5)	0.36
	5	1.1 (0.9, 1.4)		11.1 (5.5, 32.2)		15.4 (5.7, 30.6)	
	20	3.9 (3, 5.3)		38.5 (24.6, 81.1)		10.1 (6.5, 13.6)	
4OHNDtam	1	2.6 (1.8, 3.1)	<0.0001	31.3 (17.7, 52)	<0.0001	11.9 (6.8, 23.9)	0.63
	5	9 (6.7, 12.3)		80.3 (27, 201.1)		10.9 (3.7, 15.4)	
	20	37.5 (25.5, 43.9)		339.6 (157.7, 507.8)		9.7 (6.1, 27.4)	
NDtam	1	12.2 (9.1, 16.7)	<0.0001	68.6 (37.9, 107.1)	<0.0001	6.4 (3, 8.5)	0.59
	5	49.3 (41.2, 68.7)		226.8 (56.1, 457.4)		6.4 (1, 11)	
	20	167.6 (147.1, 223.9)		617.4 (348.1, 1615.9)		4.3 (2.1, 8.4)	
NDDtam	1	1.2 (1, 1.9)	<0.0001	7.6 (3.2, 11.7)	<0.0001	5.7 (3.2, 8.2)	0.61
	5	5.7 (4.4, 8.6)		21 (7.5, 41.1)		4.5 (1.4, 8.3)	
	20	24.3 (17.9, 32.2)		80.9 (28.1, 193.2)		4.5 (1.7, 8.2)	
TamNox	1	1 (1, 1.5)	<0.0001	1.2 (1, 1.7)	0.0002	1.1 (1, 1.6)	<0.0001
	5	5 (3.1, 7.9)		2.1 (1.2, 4.9)		0.5 (0.2, 1.3)	
	20	19.8 (11.2, 37.8)		2.5 (1.6, 6.7)		0.2 (0.1, 0.2)	

^a^Non-parametric Wilcoxon test was used to evaluate differences between treatment groups receiving 1, 5 or 20 mg tamoxifen

**Table 3:** Associations between serum and tissue levels of tamoxifen and its metabolites

	*n*	Serum					
		Tamoxifen	4OHtam	4OHNDtam	NDtam	NDDtam	TamNox
		R^a^	R	R	R	R	R
Tumor tissue	63	0.688	0.594	0.645	0.631	0.679	0.492

^a^R: Spearman rank correlation coefficient

*P* < 0.001 for all R values

We obtained data regarding adverse effects, such as hot flashes, sweats, and night sweats using the Menopause-Specific Quality of Life questionnaire [30]. Baseline symptom questionnaires were available for 84 patients. Although not significant, the serum levels of tamoxifen, 4OHtam, 4OHNDtam, NDtam, and NDDtam were higher in patients who experienced worsening of menopausal related symptoms from baseline compared to patients without change of these symptoms (Table 4) irrespective of treatment group.

**Table 4:** Serum concentrations of tamoxifen and metabolites in patients with or without adverse effects

	No adverse effects (*n* = 36)	Experienced adverse effects (*n* = 11)
	Median (Q1, Q3) (ng/ml)	Median (Q1, Q3) (ng/ml)
Tamoxifen	15.11 (5.61, 58.87)	38.19 (25.23, 70.97)
4OHtam	0.76 (0.25, 2.94)	1.68 (1.3, 2.95)
NDtam	31.67 (11.01, 128.31)	72.69 (55.67, 140.64)
4OHNDtam	8.36 (2.28, 29.43)	17.73 (8.05, 37)
NDDtam	4.57 (1, 17.07)	10.14 (4.9, 19.47)
TamNox	2.04 (1, 12.5)	9.12 (6.74, 10.49)

Worsening of hot flashes, sweats and night sweats. Data obtained from all patients who filled in the Menopause-Specific Quality of Life questionnaire irrespective of dosing groups

**Fig. 1:**
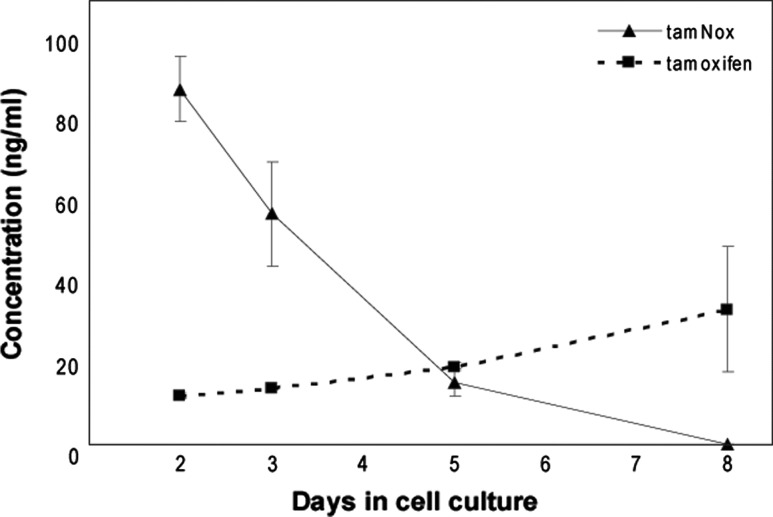
Concentrations of tamoxifen and tamNox in the growth medium of MCF-7 breast cancer cells treated with tamNox. The level of tamNox was measured at day 2, 3, 5, and 8 after administration. The figure shows the mean ± SD of six-well plates. The results are representative of two independent experiments

To investigate the in vitro metabolism of tamNox, ER+ MCF-7 breast cancer cells were treated with tamNox for 8 days. During the 8 days of treatment, we observed that the levels of tamNox decreased, while the concentrations of tamoxifen increased when measured in cell culture media (Fig. 1). This trend was observed even after 2 days. On day 8, the levels of tamoxifen were 14–20 % that of the initial tamNox levels. No other tamoxifen metabolites were detected in the culture media. Thus, it appears that tamNox was reduced to tamoxifen in these ER+ MCF-7 breast cancer cells. Notably, it has been suggested that tamNox may represent a metabolic intermediate between tamoxifen and NDtam. However, in the present in vitro study NDtam was not generated.

## Discussion

The activity and adverse effects of tamoxifen may be attributed not only to the concentrations of 4OHNDtam but also to the parent drug and some of its metabolites. In the present study, we report for the first time, the distribution of 4OHtam, 4OHNDtam, and tamNox in breast cancer tissue during normal- and two low-dose regimens using a highly sensitive HPLC-MS/MS assay. We have previously presented pharmacokinetic results from this study by using an HPLC-fluorescence method [25]. However, we were not able to detect 4OHtam in several tissue samples, and 4OHNDtam and tamNox were not quantified in the previous study. In the present study, we detected 4OHtam, 4OHNDtam, and tamNox in all serum and tissue samples. In agreement with previous studies, we observed a high inter-individual variability in the 4OHtam and 4OHNDtam concentrations in serum and tissues in each of the dosage groups [25]. For the first time, we observed that the breast cancer tissue levels of 4OHtam and 4OHNDtam (endoxifen) were closely related to those in serum during the normal- and low-dose regimens. In contrast, the tissue-to-serum ratio of tamNox decreased with increasing tamoxifen dose.

An optimal concentration range for tamoxifen and its metabolites has not been established. However, tamoxifen dosage is based on the one-dose-fits-all approach. Tamoxifen concentrations increase with age [31–33], and liver and renal function may influence its elimination [34, 35]. Furthermore, the metabolism of tamoxifen is influenced by polymorphism of tamoxifen-metabolizing enzymes [35] and multiple interacting drugs [13, 25]. Madlensky et al*.* [36] suggested that 4OHNDtam has a concentration threshold that is effective against the recurrence of breast cancer and that approximately 80 % of those taking tamoxifen using the conventional dose of 20 mg daily reach this threshold. Also, the adverse effects of tamoxifen appears to be concentration-dependent [7] and the risk of endometrial cancer has been associated with the duration of treatment and accumulated dose [37]. The large inter-individual pharmacokinetic variability observed suggests that treatment may be improved by individualization of the dose by maintaining serum tamoxifen metabolite concentrations within a target range.

Anti-estrogenic activity of tamNox has been observed. Bates et al*.* [21] found a relative binding affinity of tamNox for ER comparable to that of tamoxifen. Furthermore they have shown that the growth of ER-positive cell line MCF-7 was inhibited to the same level by tamoxifen and tamNox and this effect was reversed by the addition of E2 to the culture medium. Neither tamoxifen nor tamNox inhibited the growth of ER negative cells.

Distribution and effects of tamNox have been poorly examined in humans. In the present study, the tissue-to-serum ratios of tamoxifen, 4OHtam, 4OHNDtam, NDtam, and NDDtam did not show any significant changes with increasing tamoxifen dose, suggesting rapid tissue distribution. However, the tissue distribution of tamNox differed from that of the other metabolites as the tissue-to-serum ratio of tamNox decreased with increasing tamoxifen dose. A rapid conversion of tamNox to tamoxifen, together with a rate-limited production of tamNox from tamoxifen may explain this. In line with this observation, Parte and Kupfer [22] observed an extremely rapid reduction to tamoxifen in liver microsomes. The reaction was catalyzed by numerous CYPs without major selectivity and was also catalyzed by several other heme-containing compounds including hemoglobin.

In the present study, we observed that MCF-7 cells also convert tamNox to tamoxifen, but no other tamoxifen metabolites were detected. This confirms that a reduction of tamNox to tamoxifen occurred in these ER+ MCF-7 breast cancer cells.

Using capillary electrophoresis combined with mass spectrometry, Carter et al*.* [38] observed that patients with macroscopic metastatic tumors had substantially higher amounts of tamNox in their urine than those patients who had no evident macroscopic metastases. They speculated whether breast cancer itself might influence tamoxifen metabolism [38].

Lu et al*.* [39] recently observed inhibition of aromatase by tamNox, NDtam, and endoxifen using microsomal incubations. However, in a study of postmenopausal women the serum levels of estrogens were positively related to the levels of tamNox during steady-state tamoxifen treatment [35]. Thus, the aromatase inhibition observed in vitro seems not to dominate in vivo.

We observed a non-significant trend toward higher levels of tamoxifen and its metabolites in subjects who experienced side effects. This is consistent with earlier studies showing that patients who experienced tamoxifen-related adverse effects have high levels of tamoxifen and/or NDtam [7, 32]. Such adverse effects may result in non-compliance, and consequently, under-treatment and an increased risk of local and distant disease recurrence [40–44].

The strong relationship observed between the concentrations of tamoxifen and its metabolites in serum and target tissues suggest that these serum concentrations provide information about the corresponding levels in target tissues. Many of the common criteria for therapeutic drug monitoring are fulfilled for tamoxifen, such as correlations between serum concentrations and its effects and adverse effects, major inter-individual variation in metabolism, difficulties in observing toxicity clinically, possibility of interactions and problems related to compliance.

One limitation of the present study is that the samples had been thawed once and stored for 6 years before analysis. However, Teunissen et al*.* [45] had observed that the stability of tamoxifen and its metabolites in serum samples is guaranteed for at least three freeze (−80 °C)/thaw cycles. In addition, the levels in this study are in agreement with our earlier results from the same samples using an HPLC method [25]. Another limitation of the present study is that the HPLC-MS/MS method used does not separate the E-isomer of 4OHNDtam from the therapeutically active Z-isomer. However, serum concentrations of tamoxifen and the demethylated and hydroxylated metabolites, with the exception of tamNox, corresponded well to their concentrations in breast cancer tissue. These measurements suggest that this is also the case for 4OHNDtam.

In conclusion, we report that breast cancer tissue levels of tamoxifen and its metabolites are related to their serum levels during normal- and low-dose regimens. In contrast, the tissue-to-serum ratio of tamNox decreased with increasing tamoxifen dose. This may be due to a fast reduction of tamNox to tamoxifen in breast cancer cells and a rate-limited production of tamNox. The levels of tamoxifen and its metabolites were non-significantly higher in serum and tumor tissue of subjects experiencing side effects compared to those who did not. Tamoxifen dosage is based on the one-dose-fits-all approach although the concentrations of tamoxifen and its metabolites vary widely between patients. An optimal concentration range for tamoxifen and its metabolites remains to be established. The strong relationship observed between the concentrations of 4OHNDtam in serum and target tissues suggest that the implementation of individualized tamoxifen dosing based on therapeutic drug monitoring may be useful as a tool to improve tamoxifen therapy.

## Abbreviations

4OHtam: 4-Hydroxytamoxifen
4OHNDtam/endoxifen: 4-Hydroxy-*N*-desmethyltamoxifen
tamNox: Tamoxifen-*N*-oxide
NDtam: *N*-desmethyltamoxifen
NDDtam: *N*-desdimethyltamoxifen
SERM: Selective estrogen receptor modulator
ER: Estrogen receptor
SULTs: Sulfotransferases
UGTs: Uridine-diphospho-glucuronosyltransferases
FMO: Flavin-containing monooxygenase
HPLC–MS/MS: High pressure liquid chromatography-tandem mass spectrometry
PR: Progesterone receptor
